# Humoral Immunity and Antibody Responses against Diphtheria, Tetanus, and Pneumococcus after Immune Effector Cell Therapies: A Prospective Study

**DOI:** 10.3390/vaccines12091070

**Published:** 2024-09-19

**Authors:** Georgios Angelidakis, Roy F. Chemaly, Pranoti V. Sahasrabhojane, Oscar Morado-Aramburo, Ying Jiang, Micah M. Bhatti, Elizabeth Shpall, Chitra Hosing, Preetesh Jain, Kris Michael Mahadeo, Fareed Khawaja, Peter Elhajj, Jennifer A. Wargo, Robert R. Jenq, Nadim J. Ajami, Partow Kebriaei, Ella J. Ariza-Heredia

**Affiliations:** 1 Departments of Infectious Diseases, Infection Control and Employee Health, Division of Internal Medicine, The University of Texas MD Anderson Cancer Center, Houston, TX 77030, USA; gangelidakis@mdanderson.org (G.A.); rfchemaly@mdanderson.org (R.F.C.); omoradoar@gmail.com (O.M.-A.); yijiang@mdanderson.org (Y.J.); fkhawaja@mdanderson.org (F.K.); pelhajj@tamu.edu (P.E.); 2 Department of Genomic Medicine, The University of Texas MD Anderson Cancer Center, Houston, TX 77030, USA; pvsahasrabhojane@mdanderson.org (P.V.S.); jwargo@mdanderson.org (J.A.W.); rjenq@coh.org (R.R.J.); najami@mdanderson.org (N.J.A.); 3 Platform for Innovative Microbiome and Translational Research, The University of Texas MD Anderson Cancer, Houston, TX 77030, USA; 4 Department of Clinical Microbiology, Division of Pathology/Lab Medicine, The University of Texas MD Anderson Cancer Center, Houston, TX 77030, USA; mmbhatti@mdanderson.org; 5 Department of Stem Cell Transplantation and Cellular Therapy, Division of Cancer Medicine, The University of Texas MD Anderson Cancer Center, Houston, TX 77030, USA; eshpall@mdanderson.org (E.S.); cmhosing@mdanderson.org (C.H.); pkebriae@mdanderson.org (P.K.); 6 Department of Lymphoma/Myeloma, Division of Cancer Medicine, The University of Texas MD Anderson Cancer Center, Houston, TX 77030, USA; pjain@mdanderson.org; 7 Department of Pediatrics, Division of Pediatric Transplant and Cellular Therapy, Duke University School of Medicine, Durham, NC 27705, USA; kris.mahadeo@duke.edu; 8 Department of Surgical Oncology, Division of Surgery, The University of Texas MD Anderson Cancer Center, Houston, TX 77030, USA

**Keywords:** tetanus, diphtheria, pneumococcus, immune effector cell therapies, humoral immunity, CAR-T

## Abstract

Patients undergoing immune effector cell therapy (IECT) are at high risk for infections. We assessed seropositivity against pneumococcus, tetanus, and diphtheria in patients before and after IECT and the patients’ response to vaccination. We enrolled patients who underwent IECT from January 2020 to March 2022. Antibody levels for diphtheria, tetanus, and pneumococcus were measured before IECT, at 1 month, and 3–6 months after. Eligible patients were vaccinated after IECT. In non-seroprotected patients, we discontinued testing. Before IECT, most patients had seroprotective antibody levels against tetanus (68/69, 99%) and diphtheria (65/69, 94%), but fewer did against pneumococcus (24/67, 36%). After IECT, all patients had seroprotective antibody levels for tetanus at 1 month (68/68) and 3–6 months (56/56). For diphtheria, 65/65 patients (100%) had seroprotective antibody levels at 1 month, and 48/53 (91%) did at 3–6 months. For pneumococcus, seroprotective antibody levels were identified in 91% (21/23) of patients at 1 month and 79% (15/19) at 3–6 months following IECT. Fifteen patients received a pneumococcal vaccine after IECT, but none achieved seroprotective response. One patient received the tetanus-diphtheria vaccine and had a seroprotective antibody response. Because some patients experience loss of immunity after IECT, studies evaluating vaccination strategies post-IECT are needed.

## 1. Introduction

Immune effector cell therapies (IECTs) including chimeric antigen receptor (CAR) T-cell therapy, CAR natural killer cell therapy, and T-cell receptor–directed immunotherapy are potent immunotherapies that have revolutionized cancer treatment by selectively targeting and eliminating cancer cells [1,2]. These therapies involve engineering the patients’ T cells or natural killer cells to target antigens expressed on cancer cells. IECT has shown remarkable clinical responses in some patients with relapsed or refractory hematologic malignancies and solid tumors [2,3].

However, IECT has a substantial impact on the immune system, especially IECT targeting CD19, a type-1 transmembrane glycoprotein widely expressed on B-cells. This can have long-term implications for humoral immunity because CAR T cells target and can eliminate CD19+ B cells, leading to prolonged B cell depletion [4]. B-cell depletion can lead to hypogammaglobulinemia and, along with reported prolonged cytopenia, can result in an increased risk of infections [5,6,7,8,9].

Antimicrobial therapy constitutes a valuable tool for preventing infections after IECT. However, in the long-term for patients who have successfully undergone IECT, vaccination is of utmost importance. Currently, there are limited data available regarding the efficacy of immunization after IECT including which vaccines would be beneficial, their effectiveness, and the appropriate timing for vaccination. Recent studies have shown inadequate seroprotective antibody titers in CAR T-cell therapy recipients against vaccine-preventable diseases such as mumps, hepatitis A virus, hepatitis B virus, *Haemophilus influenzae* type b, *Streptococcus pneumoniae*, *Bordetella pertussis*, and SARS-CoV-2 [10,11,12].

Despite the increasing use of IECTs, given the lack of data on vaccination after IECTs, there are currently no formal guidelines or recommendations. To address this gap, the goal of this study was to determine the rate of antibody seropositivity against vaccine antigens for tetanus, diphtheria, and pneumococcus before and after IECT as well as to investigate factors associated with retained immunity and evaluate the humoral immune response to tetanus-diphtheria and pneumococcal vaccines after IECTs.

## 2. Materials and Methods

### 2.1. Patients, Study Design, and Study Assessments

We conducted a prospective, observational, single-center cohort study of children and adults with any type of cancer including hematologic and solid tumor malignancies for which they received commercial or investigational IECT such as CAR T-cell therapy, CAR natural killer cell therapy, and T-cell receptor-directed immunotherapy. We enrolled patients between January 2020 and March 2022 at the MD Anderson Cancer Center before they underwent IECT.

**Figure 1:**
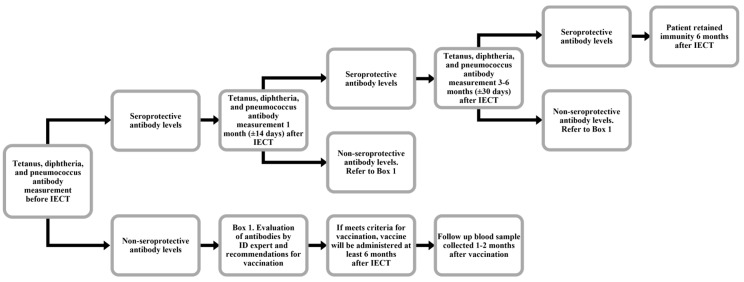
Study plan for antibody blood testing, Abbreviations: IECT, immune effector cell therapy.

After patient enrollment, baseline blood samples were collected to immunoglobulin G (IgG) concentration for diphtheria (diphtheria toxoid IgG antibody, serum), tetanus (tetanus toxoid IgG antibody, serum), and *Streptococcus pneumoniae* (total *Streptococcus pneumoniae* 23-valent serotype IgG antibodies, serum) within 4 months prior to starting lymphodepleting chemotherapy for IECT. Antibody assays for diphtheria, tetanus, and pneumococcus were performed by Mayo Clinic Laboratories at Rochester. For patients with seroprotective antibody levels at the baseline according to our laboratory standards, a follow-up blood draw was performed at 1 month (±14 days) after IECT, and again at 3–6 months (±30 days) after IECT if the 1-month antibody test result remained at seroprotective levels. Patients with antibody titers below the level of seroprotection were not subjected to further blood draws or evaluation. As per protocol, results of the serologic tests were shared with the primary clinical providers after the 3–6 months post-IECT time point and determined eligibility for the respective vaccine. As this was not an interventional study, the decision of vaccination after IECT was conducted by the primary oncologist if it was determined that the patients would be eligible for vaccine given their clinical status. Patients who received the pneumococcal and/or tetanus-diphtheria vaccine at 6 to 12 months after IECT were tested for antibody responses 1–2 months after vaccination (Figure 1).

Patients who received pneumococcal or tetanus-diphtheria vaccines as part of a trial and/or had received immunoglobulins within 2 months from the date of lymphodepleting chemotherapy for IECT were excluded during the screening process.

### 2.2. Interpretations of the Tests

Diphtheria and tetanus antibody tests were performed using the enzyme-linked immunosorbent assay at the Mayo Clinic laboratory. As per laboratory definition, patients with diphtheria IgG antibody values ≥ 0.01 IU/mL and tetanus IgG antibody values ≥ 0.01 IU/mL were considered to have seroprotective antibody levels, for the purposes of the study [13].

The test for *Streptococcus pneumoniae* IgG antibodies was performed using microsphere photometry. The 23 pneumococcal serotypes and their normal values were evaluated [14]. Patients who had antibody concentrations greater than or equal to the reference value for at least 50% of the serotypes were considered to have seroprotective antibody levels for the purposes of the study, using the Mayo Clinic laboratory reference criteria [14,15,16]. For the pneumococcal vaccine, a vaccine response was defined as at least a 2- to 4-fold increase in titers for at least 50% of the serotypes after vaccination compared with before vaccination [15,17]. For the tetanus-diphtheria vaccine, a vaccine response was defined as the achievement of serum antibody concentration > 0.1 IU/mL for tetanus and diphtheria [18]. We collected data about intravenous immunoglobulin (IVIG) administration before titers were evaluated to help determine its impact on antibody titers.

### 2.3. Statistical Analysis

Data were extracted from medical records and electronic databases from 6 months before through to 1 year after IECT. Descriptive statistics were used to summarize the patient data. Continuous variables were summarized using mean or median and range or interquartile range, and categorical variables were summarized using frequency and percentage. Continuous variables were compared using the Wilcoxon rank-sum test, and categorical variables were compared using the chi-square or Fisher’s exact test, as appropriate. Antibody titers for diphtheria, tetanus, and pneumococcus at each time point were displayed using a box plot or bar graph. Given that our research aim was to investigate the immediate and longer-term effects of IECT on antibody seropositivity against vaccine antigens, the primary focus of the analysis was to examine the differences in antibody titers between specific time points. Therefore, each specific antibody at 1 month and 3–6 months after IECT were compared with those collected before IECT using the Wilcoxon signed-rank test. Correlation between the total IgG levels and levels of IgG specific to tetanus, diphtheria, and the pneumococcus-specific serotypes were evaluated using Spearman rank correlation, respectively. All tests were 2-sided with a significance level of 0.05. The statistical analyses were performed using SAS version 9.4 (SAS Institute Inc., Cary, NC, USA).

### 2.4. Study Oversight

The study was approved by the Institutional Review Board of The University of Texas MD Anderson Cancer Center, and all enrolled patients signed an informed consent document before any study activity was undertaken.

## 3. Results

**Figure 2:**
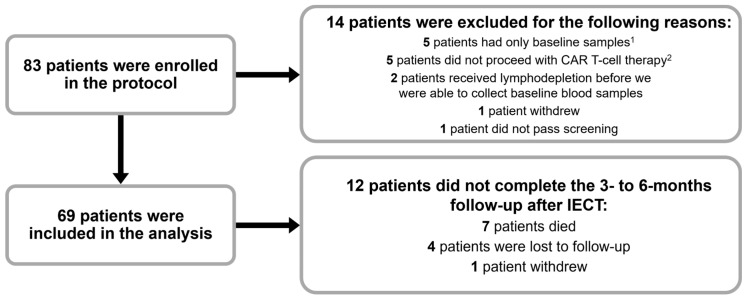
Patient enrollment flowchart, Abbreviations: CAR, chimeric antigen receptor. ^1^ Two patients died before receiving immune effector cell therapy, 2 patients died right after IECT, and 1 patient went to hospice after IECT. ^2^ One patient had apheresis but ended up not being a candidate for IECT, 1 patient did not receive IECT because all protocols were shut down due to the COVID-19 pandemic, 1 patient had two failed apheresis attempts, 1 patient had a CAR T-cell therapy manufacturing failure, and 1 patient was in remission.

A total of 83 patients were enrolled in the study, and 14 patients were excluded from the analysis for various reasons (Figure 2). Of the 69 patients included in the analysis, 42 (61%) were men, 49 (71%) had non-Hodgkin lymphoma, and the mean age was 57 years old (the age ranged between 16 to 84 years old). All patients in the analysis had received the tetanus-diphtheria vaccine before enrollment, and 28 patients (41%) received the pneumococcal vaccine (Table 1).

**Figure 3:**
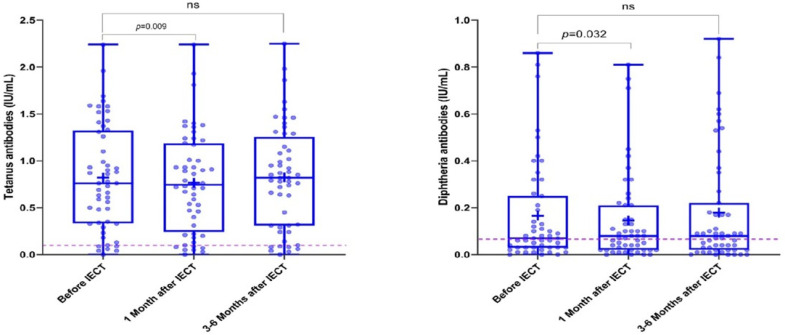
Antibody titers against tetanus and diphtheria during the study period. The left graph represents a statistically significant decrease in tetanus antibody titers between before immune effector cell therapy (IECT) and 1 month after IECT time points (*p* = 0.009). The right graph represents a statistically significant decrease in diphtheria antibody titers between before immune effector cell therapy (IECT) and 1 month after IECT time points (*p* = 0.032). The difference between the time points before IECT and 3–6 months after IECT was not statistically significant (ns).

The number of patients who had seroprotective levels of antibodies against tetanus was 68/69 (99%) before IECT, 68/68 (100%) 1 month after IECT, and 56/56 (100%) 3–6 months after IECT. The number of patients who had seroprotective levels of antibodies against diphtheria was 65/69 (94%) before IECT, 65/65 (100%) 1 month after IECT, and 48/53 (91%) 3–6 months after IECT (Table 2). Antibody titers against tetanus and diphtheria decreased from baseline to 1 month after IECT (Figure 3) but recovered on the subsequent measurement. In addition, low total IgG values were correlated with decreased levels of tetanus- and diphtheria-specific IgGs (Supplementary Figure S1), and 50% of the pneumococcus-specific serotypes including serotypes 1B, 6B, 10A, 12F, 14, 15B, 17F, 19F, 20, 22F, and 23F at 3–6 months after IECT (Supplementary Table S1).

**Figure 4:**
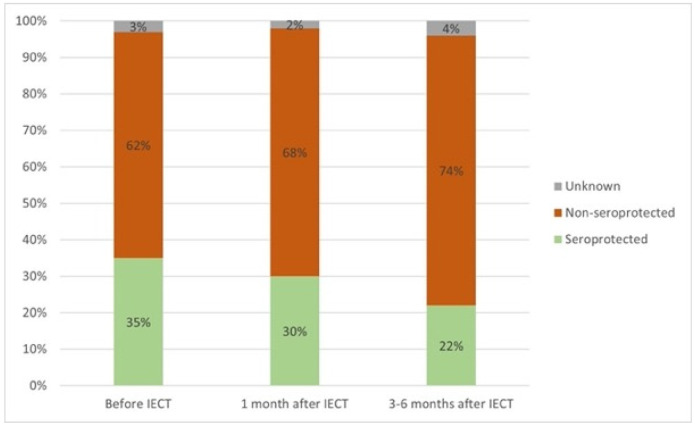
Percentage of patients with antibody titers against pneumococcus below and above the seroprotective levels at the time points before immune effector cell therapy (IECT), 1 month after IECT, and 3–6 months after IECT.

**Figure 5:**
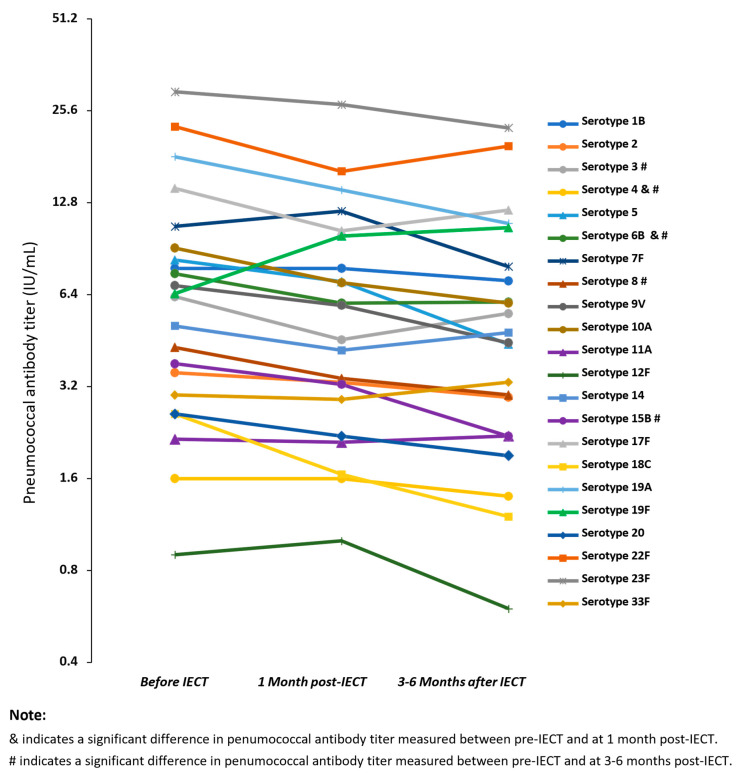
The figure represents the median immunoglobulin G (IgG) antibody titer concentrations for the Streptococcus pneumoniae IgG antibodies at 3 time points: Before immune effector cell therapy (IECT), 1 month after immune IECT, and 3–6 months after IECT.

The number of patients who had seroprotective levels of pneumococcal antibodies was 24/67 (36%) before IECT, 21/23 (91%) 1 month after IECT, and 15/19 (79%) 3–6 months after IECT (Figure 4). Of the 28 patients with a documented history of pneumococcal vaccination before enrollment, 9/28 (32%) had seroprotective antibody levels against pneumococcus at baseline. The rest of the patients with seroprotective antibody levels against pneumococcus did not have documentation of vaccination. Immunoglobulin levels were higher in patients who had seroprotective levels of pneumococcal antibodies compared with patients who had non-seroprotective antibody levels, but this difference was not statistically significant. In most patients, a further decrease in the levels of most of the 23 serotypes was observed at 3–6 months after IECT. That decrease was statistically significant for serotypes 3, 4, 6B, 8, and 15B (Figure 5).

Of the 54 patients who did not have seroprotective levels of pneumococcal antibodies at the baseline or after IECT, 15 (28%) received the pneumococcal vaccine at a median of 6 months (within a range of 4 to 12 months) after IECT and had follow-up serologic testing and 1 (2%) patient received the vaccine but was lost to follow-up. Among the 15 patients who were vaccinated and had follow-up data, 11 received a pneumococcal conjugate vaccine with 13 serotypes (PCV13), 2 received PCV13 followed by the pneumococcal polysaccharide vaccine (PPSV23), 1 patient received a pneumococcal conjugate vaccine with 20 serotypes (PCV20), and 1 received a PPSV23 vaccine. None of the 15 patients had an adequate immune response to the pneumococcal vaccine as measured by antibody response ~4 weeks after vaccination. One patient received the tetanus-diphtheria vaccine and had an adequate immune response. Of interest, the patient that had a positive response to tetanus diphtheria did not have an adequate response to the pneumococcal vaccine. Although the limited number did not allow for a definitive conclusion, these results reinforce the poor immunogenicity of the pneumococcal polysaccharide vaccine in immunocompromised patients.

Thirty-eight patients (70%) did not receive the pneumococcus or tetanus-diphtheria vaccine as recommended during the study follow-up period for various reasons: 11 (29%) developed progressive disease and/or needed active cancer therapy, 12 (31%) were lost to follow-up, 10 (26%) died, 3 (8%) entered hospice care, and 2 (5%) refused vaccination.

Regarding immune reconstitution, laboratory tests for the patients in our analysis at 3–6 months had a mean WBC 3.0 K/μL, CD4 count of 111 cells/µL, and IgG level of 547 mg/d (Table 2). Most patients exhibited a complete absence of B-lymphocyte antigen CD19 cells, with a median CD19 absolute quantity of 0 cells/µL at each time point and up to 1 year after IECT (Table 2). There was not a difference in immune reconstitution parameters between the patient who retained and lost immunity for pneumococcal antibodies (Supplement Table S2).

Seven patients received IVIG during the study follow-up period. All patients had non-seroprotective levels of antibodies against pneumococcus before the IVIG infusion including one patient who also had non-seroprotective levels of antibodies against diphtheria before the IVIG infusion. Two of these patients received a PCV13 vaccine after IVIG infusion but did not mount an adequate immune response, and one of them was also vaccinated with a tetanus-diphtheria vaccine after IVIG and had an adequate immune response. The remaining five patients were not retested for pneumococcal serologies after IVIG infusion.

## 4. Discussion

In the present prospective study, we assessed the antibody levels for tetanus, diphtheria, and pneumococcus antigens in patients before and after IECT, and our findings illustrate the potential challenges and implications of vaccination in this unique patient population.

In our cohort, most patients had seroprotective levels of antibodies against tetanus and diphtheria before IECT. Regarding the pneumococcal antibodies prior to IECT, approximately two-thirds of the patients did not have seroprotective levels before IECT including the majority of patients that had previously received the pneumococcal vaccine, which is consistent with previously reported findings in the CAR T-cell therapy recipients [10,15]. Interestingly, among the patients with seroprotective levels of pneumococcal antibodies before IECT, one-fourth experienced further antibody loss after cellular therapy. Similar results about loss of immunity over time have previously been reported in hematopoietic cell transplant recipients [19,20,21], and more recently in recipients of IECT who received influenza vaccine prior to cellular therapy [22]. In addition, our analysis depicts how antibody titers against pneumococcal serotype 8 in particular significantly decreased after IECT. It is important to mention that a recent publication reported that invasive pneumococcal disease caused by non-vaccine serotypes including serotype 8 is increasing in North America [23]. The above findings highlight the importance of monitoring antibody levels after IECT and the importance for preventive vaccines in this at risk population.

With regard to tetanus and diphtheria, our findings align with surveillance data and recent publications, which have shown similar rates of seroprotective antibodies against tetanus and diphtheria in both the general population and cancer patients who have undergone CAR T-cell therapies [10,24], Furthermore, we observed a correlation between low IgG values and decreased antibody levels for tetanus and diphtheria at 3–6 months after IECT.

It is important to note that our reference laboratory used a very conservative threshold for seroprotection for tetanus and diphtheria of 0.01 IU/m, whereas some publications considered titers between 0.01 and 0.099 IU/mL as basic protection or undetermined seroprotective levels [25,26,27,28], and others have defined >0.1 IU/mL as the seroprotective determined by the standard toxin neutralization method [27,29,30,31]. If we were to use the >0.1 IU/mL cutoff for tetanus/diphtheria for patients in our study who completed the 3- to 6-month follow-up after IECT, most patients (88%) had seroprotective levels of antibodies against tetanus, but 51% would not have met the criteria for seroprotective levels of antibodies against diphtheria, and would therefore benefit from Td vaccination after cellular therapy.

In terms of vaccine response, in our cohort, pneumococcal vaccination between 4 months and 1 year after IECT did not yield seroprotective antibody responses. Factors such as low white blood cell and CD4 counts at 3–6 months after IECT as well as low B-cell CD19 likely influenced the patient’s poor response to the pneumococcal vaccine. Recent studies evaluating vaccine immunogenicity in CAR T-cell therapy recipients have also highlighted the impairment of humoral immunity following these therapies and its impacts on vaccine response [32,33,34] Walti et al. recently evaluated the immunogenicity of influenza vaccination in CAR T-cell therapy recipients, and they reported an antibody response to >1 influenza vaccine strain in 40% of patients who received the vaccine prior to CAR T-cell therapy, compared with 31% of those who received the vaccine after therapy [22]. A recent publication from the Moffitt Cancer Center also described low immunogenicity for PCV13 in patients after IECT [35].

Current recommendations on schedules for vaccination after IECT are derived from center protocols and expert opinion and are based on data on stem cell transplant patients [30,36]. More data specifically on vaccine response for patients after IECT are needed to help determine the best strategies for immune protection. Recently, Gössi et al. reported that patients who received two doses of COVID-19 vaccine after CAR T-cell therapy had very low seropositivity (23%), with an improvement in antibody titers and anti-spike protein IgG after a third and fourth booster [32]. This strategy has been used in other populations to improve the levels of seroprotection, thus it makes sense to use a similar strategy in IECT recipients [32]. Furthermore, some publications have shown an association between lower rates of seroconversion with a shorter interval of cellular therapy to vaccination [37]. Further evaluation of the best timing for vaccination as well as the value of immune reconstitution parameters and cutoff values used to help guide vaccine recommendations in patients after IECT are needed [33].

Our study had some limitations. First, the sample size was relatively small because several patients died during the follow-up period, which may limit the generalizability of the findings to larger cohorts with different types of IECT. Second, the study lacked a control group of cancer patients who did not receive IECT. Without a control group, it is challenging to determine whether the observed changes in vaccine response were specifically attributed to IECT or could have been influenced by other confounders such as the underlying malignancies and/or previous line of therapies. Third, the number of patients who received a vaccine was relatively small, and the majority of patients did not recall timing or type of previous immunizations, which restricted our ability to determine the immune responses to vaccination after IECT. Finally, our study focused on evaluating humoral immunity, but other aspects of the immune system, such as cellular immune responses, were not investigated.

## 5. Conclusions

The present study provides valuable insights into humoral immunity over time to tetanus, diphtheria, and pneumococcus in cancer patients undergoing IECT. We showed that the levels of antibody titers against pneumococcus declined after IECT. Moreover, patients who received the pneumococcal vaccine after IECT were not able to elicit a good immune response to standard doses of the pneumococcal conjugated vaccine or conjugated vaccine, followed by one dose of the polysaccharide vaccine from 4 months to up to 1 year after IECT. These findings underscore the importance of monitoring and optimizing vaccination strategies in cancer patients undergoing IECT to enhance protective immune responses in vaccine-preventable infections in well-designed clinical trials. Further research with larger cohorts and different IECT types is warranted to validate and expand on our findings and determine the predictors of vaccine responses based on biomarkers, timing, and number of vaccine doses after IECT.

## Figures and Tables

**Table 1:** Demographic and clinical characteristics of patients included in our analysis (n = 69).

Characteristic	No. Patients (%)
Mean age (range), years	57 (16–84)
Sex	
Female	27 (39)
Male	42 (61)
Comorbidities	
Hypertension	26 (38)
Diabetes mellitus type 1 or 2	14 (20)
Chronic obstructive pulmonary disease	2 (3)
Coronary artery disease	1 (1)
Chronic kidney disease stages 3–5	4 (6)
Primary cancer diagnosis	
Non-Hodgkin lymphoma	49 (71)
Multiple myeloma	6 (9)
Hodgkin lymphoma	5 (7)
Chronic lymphocytic leukemia	1 (1)
Acute myeloid leukemia	2 (3)
Acute lymphoblastic leukemia	1 (1)
Solid cancer ^1^	5 (7)
History of hematopoietic cell transplant	13 (19)
Autologous	11/13 (85)
Allogeneic	2 (15)
Pneumococcal vaccination after hematopoietic cell transplant	9/13 (69)
Pneumococcal vaccination before enrollment on this study	28 (41)
Diphtheria and tetanus vaccination before enrollment	69 (100)
Lymphodepleting chemotherapy	
Fludarabine, cyclophosphamide	61 (88)
Fludarabine, bendamustine	4 (6)
Fludarabine, cyclophosphamide, rituximab	4 (6)
Type of immune effector cell therapy	
Anti-CD19 CAR T-cell therapy	47 (68)
BCMA CAR T-cell therapy	5 (7)
CAR natural killer cell therapy	4 (6)
Peptide-HLA T-cell receptor therapy	5 (7)
CD30 CAR T-cell therapy	4 (6)
CLL-1 CAR T-cell therapy	2 (3)
CD4 CAR T-cell therapy	1 (1)
CD70 CAR T-cell therapy	1 (1)
Cancer status within 1 year after immune effector cell therapy	
Remission	32 (46)
Progression/relapse	37 (54)
Mean time to relapse (IQR), months	4 (1–6)
Death within 1 year after immune effector cell therapy	26 (38)

Abbreviations: CAR, chimeric antigen receptor; BCMA, B-cell maturation antigen; IQR, interquartile range. ^1^ Three patients had sarcoma, one patient had colon cancer, and one patient had anal cancer.

**Table 2:** Laboratory test results among the patients in our analysis before, 1 month after, and 3–6 months after immune effector cell therapy (IECT).

Laboratory Test ^1^	Time Point		
	Before IECT	1 Month after IECT	3–6 Months after IECT
Tetanus antibodies, no. (%)			
Seroprotected	68/69 (99)	68/68 (100)	56/56 (100) ^2^
Non-seroprotected	1/69 (1)	-	-
Diphtheria antibodies, no. (%)			
Seroprotected	65/69 (94)	65/65 (100)	48/53 (91) ^2^
Non-seroprotected	4/69 (6)	0 (0)	5/53 (9) ^2^
Pneumococcal antibodies, no. (%)			
Seroprotected	24/67 (36) ^3^	21/23 (91) ^4^	15/19 (79) ^5^
Non-seroprotected	43/67 (64) ^3,6^	2/23 (9) ^4^	4/19 (21) ^5^
Mean (IQR) CD4 count, cells/µL	395 (146–605)	180 (38–162)	111 (45–169)
Mean (IQR) IgG, mg/dL	667 (411–768)	532 (356–623)	547 (391–665)
Mean (IQR) white blood cell count, K/μL	6.8 (3.5–8.0)	3.0 (1.8–4.0)	3.6 (1.9–4.4)
Mean (IQR) absolute neutrophil count, K/μL	4.2 (2.1–6.0)	2.0 (0.9–2.6)	2.3 (0.9–2.9)
Mean (IQR) absolute lymphocyte count, K/μL	1.6 (0.4–1.2)	0.6 (0.2–0.7)	0.7 (0.3–0.8)
IVIG, no.		1 ^7^	6 ^8^
Median (IQR) CD19 absolute count, cells/μL	0 (0–6.5) ^9^	0 ^10^	0 (0–2.5) ^11^

Abbreviations: IQR, interquartile range; IgG, immunoglobulin G; IVIG, intravenous immunoglobulin. ^1^ Serologic tests for tetanus, diphtheria, and pneumococcus were performed on all patients before IECT. Only those with seroprotective antibody levels were re-tested at 1 month and/or 3–6 months after IECT. ^2^ Seven patients died, four were lost to follow-up, and one withdrew from the study. ^3^ Two patients had previously received the pneumococcal vaccine but were not able to undergo pneumococcal serologic testing before IECT. We tested them 1 month after IECT, and both had non-seroprotective pneumococcal antibody levels. ^4^ One patient withdrew from the study. ^5^ Two patients died before reaching the 3- to 6-month follow-up after IECT. ^6^ Seventeen patients of the 43 with non-seroprotective pneumococcal antibody levels at the baseline had previously received the pneumococcal vaccine. ^7^ One patient received IVIG after IECT but before the 1-month post-IECT serologic testing. ^8^ Four patients received IVIG between the 1-month and 3- to 6-month post-IECT follow-up, and two patients received IVIG between the 6-month post-IECT follow-up and vaccination. ^9^ Data from 13 patients. ^10^ Data from 43 patients. ^11^ Data from 37 patients.

## Data Availability

Data is contained within the article and Supplementary Materials. The data reported in this article may be commercially sensitive and not publicly available. To the extent allowed, the authors will provide access to deidentified participant-level data underlying the data presented in this article to researchers who provide a methodologically sound proposal for academic purposes to interpret, verify and extend research in the article that does not violate privacy, data encumbrance, intellectual property or other legal, regulatory, or contractual confidentiality obligations. Data provided will be subject to a data use agreement. Researchers should contact the corresponding author when applying for data access. Use of data will be restricted to the agreed purpose.

## Supplementary Materials

The following supporting information can be downloaded at: https://www.mdpi.com/article/10.3390/vaccines12091070/s1, Figure S1: Correlation between IgG values and tetanus- and diphtheria-specific IgGs at 3–6 months after IECT; Table S1: Analysis of correlation between pneumonia antibodies and IgG at 3–6 months after immune effector cell therapy; Table S2: Laboratory values of patients with seroprotective and non-seroprotective levels of pneumococcal antibodies at 3–6 months after immune effector cell therapy.
