# Supplementary material for: Humoral Immunity and Antibody Responses against Diphtheria, Tetanus, and Pneumococcus after Immune Effector Cell Therapies: A Prospective Study
Source: Vaccines (Basel). 2024 Sep 19;12(9):1070. doi: 10.3390/vaccines12091070 (PMC11436035; doi:10.3390/vaccines12091070)
Supplement: Supplementary file 1 [file vaccines-12-01070-s001.zip › vaccines-3146658-supplementary.pdf]

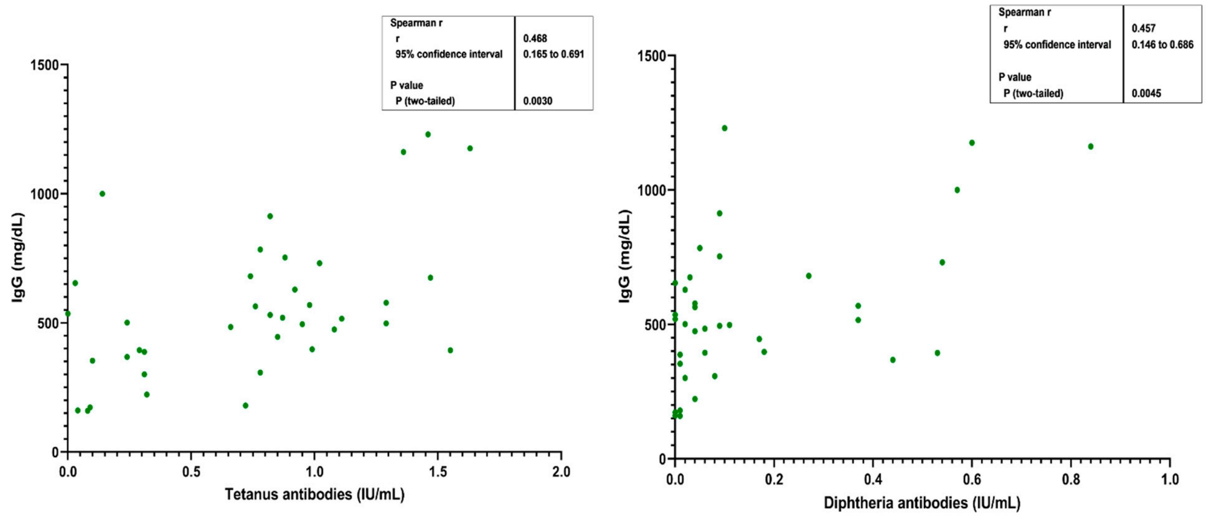

Supplementary Figure S1. Correlation between IgG values and tetanus- and diphtheria-specific IgGs at 3-6 months after IECT.

Supplementary Table S1. Analysis of correlation between pneumonia antibodies and IgG at 3-6 months after immune effector cell therapy.

Variables	Coefficient of correlation with IgG	p-value
S. pneumoniae 1B Ab	0.66	0.009
S. pneumoniae 2 Ab	0.47	0.09
S. pneumoniae 3 Ab	0.41	0.15
S. pneumoniae 4 Ab	0.38	0.18
S. pneumoniae 5 Ab	0.25	0.39
S. pneumoniae 6B Ab	0.62	0.018
S. pneumoniae 7F Ab	0.54	0.055
S. pneumoniae 8 Ab	0.37	0.19
S. pneumoniae 9N Ab	No data available for S. pneumoniae 9N Ab	
S. pneumoniae 9V Ab	0.52	0.059
S. pneumoniae 10A Ab	0.69	0.006
S. pneumoniae 11A Ab	0.13	0.65
S. pneumoniae 12F Ab	0.70	0.005
S. pneumoniae 14 Ab	0.75	0.002
S. pneumoniae 15B Ab	0.57	0.034
S. pneumoniae 17F Ab	0.69	0.007
S. pneumoniae 18C Ab	0.20	0.49
S. pneumoniae 19A Ab	0.35	0.21
S. pneumoniae 19F Ab	0.62	0.019
S. pneumoniae 20 Ab	0.57	0.033
S. pneumoniae 22F Ab	0.74	0.003
S. pneumoniae 23F Ab	0.86	< .001
S. pneumoniae 33F Ab	0.35	0.23

Abbreviations: IgG, immunoglobulin G; Ab, antibody; S. pneumoniae, Streptococcus pneumoniae

Supplementary Table S2. Laboratory values of patients with seroprotective and non-seroprotective levels of pneumococcal antibodies at 3-6 months after immune effector cell therapy.

	Median (IQR)		
**Variable**	**Seroprotective, n=15**	**Non-seroprotective, n=45**	***p***
Immunoglobulin G levels, mg/dL	569 (416-913)	500 (354-629)	0.12
CD4 count, cells/µL	99 (45-152)	72 (42-190)	0.93
White blood cell count, K/µL	3.3 (2.7-4.4)	3.0 (1.9-4.5)	0.48
Absolute neutrophil count, K/µL	2.03 (1.56-3.42)	1.88 (0.86-2.90)	0.29
Absolute lymphocyte count, K/µL	0.50 (0.33-0.73)	0.53 (0.32-0.86)	0.65
CD19 count, cells/µL	2 (0-46)	0 (0-0)	0.029

Abbreviations: IQR, interquartile range.
